# Chloroplast phylogenomics and divergence times of *Lagerstroemia* (Lythraceae)

**DOI:** 10.1186/s12864-021-07769-x

**Published:** 2021-06-09

**Authors:** Wenpan Dong, Chao Xu, Yanlei Liu, Jipu Shi, Wenying Li, Zhili Suo

**Affiliations:** 1 Laboratory of Systematic Evolution and Biogeography of Woody Plants, School of Ecology and Nature Conservation, Beijing Forestry University, 100083 Beijing, China; 2 State Key Laboratory of Systematic and Evolutionary Botany, Institute of Botany, Chinese Academy of Sciences, 100093 Beijing, China; 3 University of Chinese Academy of Sciences, 100049 Beijing, China; 4 Xishuangbanna Tropical Botanical Garden, Chinese Academy of Sciences, Mengla, 666303 China; 5 Institute of Forestry New Technologies, Chinese Academy of Forestry, 100091 Beijing, China

**Keywords:** *Lagerstroemia*, Chloroplast genome, Phylogeny, Divergence time

## Abstract

**Background:**

Crape myrtles, belonging to the genus *Lagerstroemia* L., have beautiful paniculate inflorescences and are cultivated as important ornamental tree species for landscaping and gardening. However, the phylogenetic relationships within *Lagerstroemia* have remained unresolved likely caused by limited sampling and the insufficient number of informative sites used in previous studies.

**Results:**

In this study, we sequenced 20 *Lagerstroemia* chloroplast genomes and combined with 15 existing chloroplast genomes from the genus to investigate the phylogenetic relationships and divergence times within *Lagerstroemia*. The phylogenetic results indicated that this genus is a monophyletic group containing four clades. Our dating analysis suggested that *Lagerstroemia* originated in the late Paleocene (~ 60 Ma) and started to diversify in the middle Miocene. The diversification of most species occurred during the Pleistocene. Four variable loci, *trnD-trnY-trnE*, *rrn16-trnI, ndhF-rpl32-trnL* and *ycf1*, were discovered in the *Lagerstroemia* chloroplast genomes.

**Conclusions:**

The chloroplast genome information was successfully utilized for molecular characterization of diverse crape myrtle samples. Our results are valuable for the global genetic diversity assessment, conservation and utilization of *Lagerstroemia.*

**Supplementary Information:**

The online version contains supplementary material available at 10.1186/s12864-021-07769-x.

## Background

Crape myrtles, the genus *Lagerstroemia* L. (Lythraceae, Myrtales), consisting of approximately 60 species, is mainly naturally distributed in Southern and Eastern Asia and Northern Australia [1–3]. Several species of *Lagerstroemia*, such as *L. floribunda*, *L. speciosa*, *L. macrocarpa*, *L. loudonii*, and *L. indica*, are planted as important ornamental trees. Crape myrtles are known for their long-lasting midsummer (more than 100 days) blooms from the tropical to the northern temperate zones. Cultivation of crape myrtles has been carried out for over 2,000 years. There are at least 500 named crape myrtle cultivars available in the U.S., Europe, and Asia [4].

Taxonomically, the genus *Lagerstroemia* was treated completely by Furtado & Srisuko [1], and the genus *Lagerstroemia* was fully revised and classified into three sections (including 53 species), i.e., (1) *L*. sect. *Sibia*, (2) *L*. sect. *Adambea*, and (3) *L*. sect. *Trichocarpidium*. After detailed analyses of the morphological characters and literature, De Wilde and Duyfjes [5] considered that four sections should be divided in *Lagerstroemia*: (1) *L.* sect. *Lagerstroemia*, (2) *L.* sect. *Parviflora*, (3) *L.* sect. *Adambea*, and (4) *L.* sect. *Trichocarpidium*. Several morphological character states have proven to be useful for the determination of *Lagerstroemia* [2, 5], such as the position, size, color, and auricles of flowers; the size, valves, and surface of fruits; the bark of the trunk, and the length of stamens. On this basis, some new taxa in *Lagerstroemia* have been subsequently described; during botanical surveys, several new crape myrtle taxa (species and variety) were found in Thailand, Vietnam, Cambodia and Laos [2, 5–7]. However, several plants are still known only from herbarium specimens. There are 115 *Lagerstroemia* name records in the Plant List database (http://www.theplantlist.org/), and half of the taxonomic status of the name remains unresolved.

A few phylogenetic studies have been conducted on *Lagerstroemia*, but the interspecific relationships in this group remain controversial. Phylogenetic relationships within Lythraceae based on chloroplast genic regions (*rbcL, trnL-F, psaA-ycf3*) plus the ITS region showed *Lagerstroemia* was sister to *Duabanga* and strongly supported the monophyly of the genus [8, 9]. The phylogenic relationships within *Lagerstroemia* have been poorly defined overall using several chloroplast markers and/or the ITS and gene regions of the ubiquitin-proteasome system [10, 11]. The poor phylogenetic resolution in previous studies resulted from limited amounts of DNA sequence data available and the low genetic variation in the chosen molecular markers, likely due to this group’s recent origin and rapid radiation.

Chloroplast genomes have proven to be powerful tools for studying phylogenetic relationships in related species because of their small size, high copy number, uniparental inheritance, and conserved gene content and arrangement [12–14]. In recent years, the chloroplast genomes have been sequenced and characterized for species identification and phylogenetic study [15–17]. However, due to sparse taxon sampling in previous studies, the phylogenetic relationships within *Lagerstroemia* are still unclear.

A robust phylogeny of *Lagerstroemia*, including more representative species and a large amount of genetic markers, is essential for understanding the evolutionary history, breeding of new cultivars and conservation of crape myrtle germplasm resources. In this study, we sequenced 20 chloroplast genomes of *Lagerstroemia* samples using next-generation sequencing (NGS). The aims of this study were: (i) to deepen our understanding of chloroplast genome evolution of *Lagerstroemia*, (ii) to reconstruct the robust phylogenetic relationship of *Lagerstroemia*, and (iii) to reveal the divergence times involving this genus.

## Results

### Characteristics of *Lagerstroemia* chloroplast genomes

**Fig. 1:**
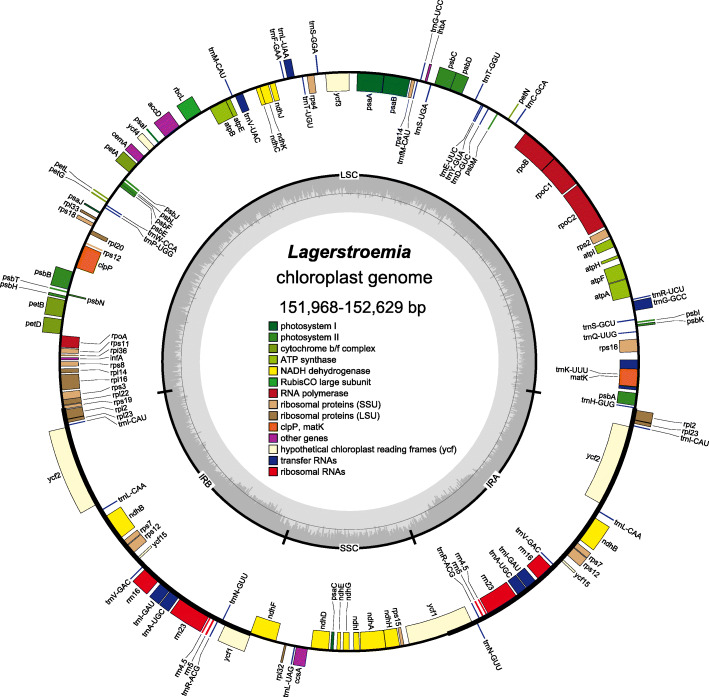
General chloroplast genome map of *Lagerstroemia*. Specific sizes for the chloroplast genomes of each species are presented in Table 1. Genes drawn outside of the map circle are transcribed clockwise, while those drawn inside are transcribed counterclockwise. Genes belonging to different functional groups are color-coded. The darker gray in the inner circle corresponds to GC, while the lighter gray corresponds to AT content

The complete chloroplast genomes of the 20 newly sequenced *Lagerstroemia* species ranged in length from 151,968 bp (*L*. *guilinensis*) to 152,629 bp (*L*. *speciosa*) (Table 1). All chloroplast genomes had the four typical conjoined structures, including the LSC and SSC regions separated by two IR regions (Fig. 1). The LSC regions ranged from 83,809 bp (*L. guilinensis*) to 84,188 bp (*L. speciosa*) and accounted for 55.20–55.26 % of the total length. The SSC regions varied between 16,729 bp (*L. anhuiensis* and *L. glabra*) and 16,920 bp (*L*. sp. 03) and accounted for 11.00–11.11 % of the total length. The IR regions ranged from 25,625 bp (*L. caudata*, *L. excelsa*, *L. fauriei*, *L. glabra*, *L. guilinensis*, *L. indica* and *L.* sp. 03) to 25,804 bp (*L*. *speciosa*) and accounted for 16.83–16.91 % of the total length. A total of 112 unique genes were detected in the chloroplast genomes of the 20 *Lagerstroemia* species, including 78 coding genes, 30 tRNA genes and 4 rRNA genes (Fig. 1; Table 1). GC content ranged from 37.6 to 37.7 %. The gene organization, gene order and GC content were highly identical and similar to those of other higher plants (Fig. 1). The overall chloroplast genomic structure, including gene number and gene order, was well-conserved.

**Table 1:** Characteristics of newly sequenced plastomes

Species	LSC length (bp)	IR length (bp)	SSC length (bp)	Plastome size (bp)	GC content (%)	Gene number	Protein coding genes	tRNA	rRNA
*L. anhuiensis*	84,058	25,631	16,729	152,049	37.6 %	112	78	30	4
* L. calyculata*	84,008	25,726	16,798	152,258	37.6 %	112	78	30	4
* L. caudata*	84,025	25,625	16,919	152,194	37.6 %	112	78	30	4
* L. excelsa*	84,047	25,625	16,917	152,214	37.6 %	112	78	30	4
* L. fauriei*	83,920	25,625	16,904	152,074	37.6 %	112	78	30	4
* L. fauriei*	83,919	25,625	16,904	152,073	37.6 %	112	78	30	4
* L. floribunda*	84,000	25,716	16,793	152,225	37.7 %	112	78	30	4
* L. glabra*	84,026	25,625	16,729	152,005	37.6 %	112	78	30	4
* L. guilinensis*	83,809	25,625	16,909	151,968	37.6 %	112	78	30	4
* L. indica*	84,060	25,625	16,919	152,229	37.6 %	112	78	30	4
* L. indica*	84,058	25,625	16,919	152,227	37.6 %	112	78	30	4
* L. intermedia*	83,997	25,732	16,850	152,311	37.6 %	112	78	30	4
* L. limii*	83,951	25,651	16,905	152,158	37.6 %	112	78	30	4
* L.* sp. 01	83,982	25,726	16,800	152,234	37.7 %	112	78	30	4
* L.* sp. 02	84,008	25,721	16,795	152,245	37.7 %	112	78	30	4
* L.* sp. 03	84,084	25,625	16,920	152,254	37.6 %	112	78	30	4
* L. speciosa*	84,183	25,714	16,832	152,443	37.6 %	112	78	30	4
* L. speciosa*	84,188	25,804	16,833	152,629	37.6 %	112	78	30	4
* L. tomentosa*	84,009	25,726	16,797	152,258	37.7 %	112	78	30	4
* L. villosa*	84,003	25,705	16,795	152,208	37.7 %	112	78	30	4

### cpDNA markers for *Lagerstroemia*

The whole chloroplast genome sequences of 35 *Lagerstroemia* (dataset-3) species were aligned to find the sequence variation. The alignment matrix of the chloroplast genome was 154,185 bp. We identified 2,029 variable sites (1.316 %), including 1,821 parsimony-informative sites (1.181 %) and 205 singleton sites (0.133 %). The overall sequence divergence estimated by p-distance among the 35 chloroplast genome sequences was 0.0049. The p-distance ranged from 0.0001 to 0.0080, and the number of nucleotide substitutions ranged from 22 to 1,215 between species.

**Fig. 2:**
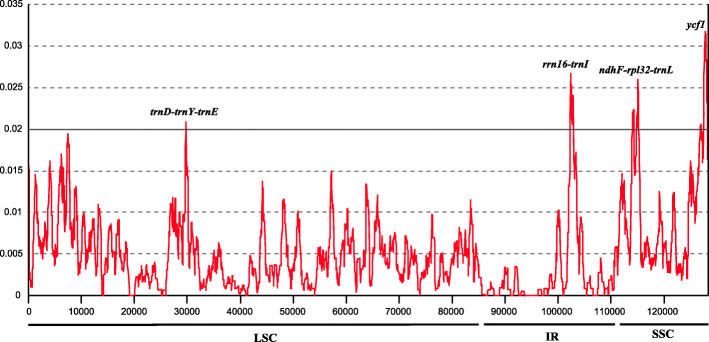
Sliding window analysis of nucleotide variability (Pi) across 35 complete chloroplast genome sequences of *Lagerstroemia*

To identify the sequence divergence hotspots, the nucleotide diversity (π) value within the slide window of 600 bp was calculated (Fig. 2). The π values varied from 0 to 0.0318, the average pi value was 0.00474, the IR region exhibited the least nucleotide diversity (0.00285), and the SSC exhibited high divergence (0.01006). Four highly variable regions (pi > 0.02), including *trnD-trnY-trnE*, *rrn16-trnI, ndhF-rpl32-trnL* and *ycf1*, were detected in the *Lagerstroemia* chloroplast genomes (Fig. 2). Among these regions, *trnD-trnY-trnE* was located in the LSC region, *rrn16-trnI* was located in the IR region, and *ndhF-rpl32-trnL* and *ycf1* were located in the SSC region. We compared the four hypervariable markers and the universal DNA barcodes (*rbcL*, *matK*, and *trnH-psbA*) in more detail (Table 2). The number of variable sites of the four markers ranged from 38 (*trnD-trnY-trnE*) to 56 (*rrn16-trnI* and *ndhF-rpl32-trnL*), whereas the universal DNA barcodes had lower divergence. The average nucleotide diversity of the four rapidly evolving regions was 0.01941, which was 2.5 times higher than that of the universal DNA barcodes. The identified variable markers had higher resolution compared with the three universal markers, based on the ML tree (Figure S1).

**Table 2:** Variability of four hyper-variable markers and the universal chloroplast DNA barcodes (*rbcL*, *matK* and *trnH-psbA*) in *Lagerstroemia*

Markers	Length	Variable sites		Information sites		Nucleotide diversity
		Numbers	%	Numbers	%	
*trnD-trnY-trnE*	1,051	38	3.62 %	36	3.43 %	0.01953
*rrn16-trnI*	1,229	56	4.56 %	51	4.15 %	0.02040
*ndhF-rpl32-trnL*	947	56	5.91 %	53	5.60 %	0.01848
*ycf1*	827	41	4.96 %	36	4.35 %	0.01890
Combine four variable markers	4,054	191	4.71 %	176	4.34 %	0.01941
*rbcL*	1,428	14	0.98 %	14	0.98 %	0.00395
*matK*	1,500	24	1.60 %	24	1.60 %	0.00636
*trnH-psbA*	138	17	12.32 %	16	11.59 %	0.06441
Combine three universial markers	3,066	55	1.79 %	54	1.76 %	0.00770

### Phylogenetic analyses

Characteristics of the six different datasets used in this study are shown in Table 3. Dataset-3 possesses the most variable and parsimony-information sites, followed by dataset-2 and dataset-4. As expected, dataset-5 (IR region) had the fewest variable and parsimony-informative sites. Dataset-1 and Dataset-2 strongly supported the monophyly of *Lagerstroemia* (BS = 100/PP = 1.0). In this study, analyses based on each dataset revealed four clades in the genus *Lagerstroemia*. Clade I was sister to Clade II, and Clade III was sister to Clade IV. Clade I included four taxa, namely, *L. siamica*, *L. intermedia*, *L. speciosa*, and *L. venusta*. Only slight differences were found between *L. speciosa* and *L. venusta*. *L. siamica* was sister to *L. intermedia*. Clade II consists of six taxa: *L. villosa*, *L. floribunda*, *L. tomentosa*, *L. calyculata*, *L*. sp. 01, and *L*. sp. 02. *L. villosa* was the first divergent species in this clade. Clade III contained three taxa: *L. fauriei*, *L. subcostata* and *L. limii*. These three taxa had longer branch on the phylogenetic tree, indicating significant divergence between each other (Fig. 4). Seven taxa are in Clade IV: *L. caudata*, *L. anhuiensis*, *L. glabra*, *L. excelsa*, *L. guilinensis*, *L. indica*, and *L*. sp. 03. *L. anhuiensis* and *L. glabra* formed a clade and showed short branch in the trees. The topology of the *Lagerstroemia* samples with high resolution was achieved based on the whole chloroplast genome sequence data (Fig. 4). Figures S2, S3, and S4 show the general decrease in resolution capacity of the topology when either the LSC, IR, or SSC region was used due to the insufficient information.

**Table 3:** Characteristics of the six different data sets

Dataset	Composition	Total number of characters (bp)	Variable sites (bp)	Parsimony-informative sites (bp)	Singleton sites (bp)
1	Nucleotide sequences of all 82 genes	71,424	628	514	114
2	Complete chloroplast genome sequences (deletion some sites according to the outgroups )	151,431	1,910	1,694	216
3	Complete chloroplast genome sequences	154,185	2,029	1,821	205
4	LSC	85,125	1,199	1,058	141
5	IR	25,998	170	156	14
6	SSC	17,065	488	434	54

### Divergence time estimate

Different fossil calibration combinations were computed to investigate the variation of estimation values of the divergence times (Table 4). We focused on the *Lagerstroemia* stem and crown nodes. The estimated age of stem-group *Lagerstroemia* showed a different pattern with younger age estimates when the fossil calibration of *Lagerstroemia patelii* (> 56 Ma, Fig. 5, Note 6) was not included. The *Lagerstroemia* stem node was 56.34 ± 4.78 Ma, and the *Lagerstroemia* crown node was 31.06 ± 2.82 Ma, obtained from the 12 fossil-calibrated analyses (Table 4).

**Table 4:** Prior setting for calibration evidence for different calibration combinations. All values are given in Ma and prior distributions are given as mean and standard deviation (stdev). Normal (N) prior distributions are applied to the secondary calibration. Lognormal (logN) prior distributions are applied to each of the fossil-calibrated nodes and are constrained to be older than the highest bound of the fossil age (offset). Priors labelled ’none’ may be interpreted as uniform, uninformative priors

Analysis	Prior distributions								Posterior distribution (95% HPD)	
	Root	Onagraceae Crown	Lythraceae Crown	Lythrum + Peplis	Sonneratia + Trapa	Lagerstroemia + Duabanga	Punica + Pemphis	Lawsonia + Rotala	Lagerstroemia Stem ~N 56.34 (4.78) Ma	Lagerstroemia Crown ~N 31.06 (2.82) Ma
	104.6 Ma	85.4 Ma	95.5 Ma	>81 Ma	>63.8 Ma	>56 Ma	>40.4 Ma	>16 Ma		
1	N 104.6 (1.0)	N 85.4 (1.0)	N 95.5 (1.0)	LogN 1.5 (1.0)	LogN 1.5 (1.0)	LogN 1.5 (1.0)	LogN 1.5 (1.0)	LogN 1.5 (1.0)	60.12 (56.20-66.27)	31.60 (14.93-49.16)
2	none	N 85.4 (1.0)	N 95.5 (1.0)	LogN 1.5 (1.0)	LogN 1.5 (1.0)	LogN 1.5 (1.0)	LogN 1.5 (1.0)	LogN 1.5 (1.0)	60.02 (56.18-65.88)	34.03 (16.83-51.40)
3	N 104.6 (1.0)	none	N 95.5 (1.0)	LogN 1.5 (1.0)	LogN 1.5 (1.0)	LogN 1.5 (1.0)	LogN 1.5 (1.0)	LogN 1.5 (1.0)	60.17 (56.22-66.28)	31.69 (15.61-50.68)
4	N 104.6 (1.0)	N 85.4 (1.0)	none	LogN 1.5 (1.0)	LogN 1.5 (1.0)	LogN 1.5 (1.0)	LogN 1.5 (1.0)	LogN 1.5 (1.0)	60.10 (56.22-66.23)	31.54 (14.69-50.03)
5	N 104.6 (1.0)	N 85.4 (1.0)	N 95.5 (1.0)	LogN 1.5 (1.0)	LogN 1.5 (1.0)	none	LogN 1.5 (1.0)	LogN 1.5 (1.0)	49.29 (28.47-69.35)	27.27 (11.18-44.64)
6	none	none	none	LogN 1.5 (1.0)	LogN 1.5 (1.0)	none	LogN 1.5 (1.0)	LogN 1.5 (1.0)	57.19 (35.71-76.81)	34.46 (17.65-52.61)
7	none	none	none	LogN 1.5 (1.0)	LogN 1.5 (1.0)	LogN 1.5 (1.0)	LogN 1.5 (1.0)	LogN 1.5 (1.0)	60.79 (56.19-68.21)	35.71 (19.35-51.33)
8	N 104.6 (1.0)	none	none	LogN 1.5 (1.0)	LogN 1.5 (1.0)	none	LogN 1.5 (1.0)	LogN 1.5 (1.0)	49.76 (26.61-71.72)	27.78 (12.63-46.89)
9	N 104.6 (1.0)	none	none	LogN 1.5 (1.0)	LogN 1.5 (1.0)	LogN 1.5 (1.0)	LogN 1.5 (1.0)	LogN 1.5 (1.0)	60.23 (56.21-66.58)	33.02 (15.88-49.95)
10	N 104.6 (1.0)	N 85.4 (1.0)	N 95.5 (1.0)	none	none	LogN 1.5 (1.0)	none	none	58.64 (56.14-62.53)	30.23 (13.90-48.40)
11	N 104.6 (1.0)	N 85.4 (1.0)	none	LogN 1.5 (1.0)	LogN 1.5 (1.0)	none	LogN 1.5 (1.0)	LogN 1.5 (1.0)	49.87 (27.83-69.97)	27.67 (12.09-46.04)
12	N 104.6 (1.0)	none	N 95.5 (1.0)	LogN 1.5 (1.0)	LogN 1.5 (1.0)	none	LogN 1.5 (1.0)	LogN 1.5 (1.0)	49.88 (29.13-69.74)	27.72 (12.80-45.44)

According to the fossil records, *Lagerstroemia* first appeared in the late Paleocene/early Eocene of the Indian subcontinent [18]. We consider the scenario including all the eight fossil calibrations as the final result (Fig. 5). The stem node of the *Lagerstroemia* was dated to 60.12 Ma (95 % highest posterior density, HPD: 56.2 − 66.27 Ma); the crown node of the *Lagerstroemia* was dated to 31.6 Ma (95 %HPD: 14.93 − 49.16 Ma). Four clades diverged approximately 19.01 Ma (95 %HPD: 5.95 − 34.17 Ma) and 11.08 Ma (95 %HPD: 2.58 − 25.28 Ma), respectively, between clades I/II and III/IV. Diversification with this genus occurred over a short time period, approximately 5.27 Ma.

## Discussion

### Informative indicated chloroplast markers for *Lagerstroemia*

Our results indicate that the mutation patterns of the chloroplast genomes were not uniform. As a whole, the single-copy region possesses a higher divergence than the IR region, and the mutation events of SNPs and indels were not random, but instead were clustered as “mutation hotspots” or “highly variable regions”. These results are generally consistent with those from other studies involving chloroplast genomes. Previous phylogenetic studies of *Lagerstroemia* mainly used the universal chloroplast loci (*rbcL*, *matK*, and *trnH-psbA*) and the ITS, but these did not provide a good resolution of the phylogenetic relationship in this genus [11]. Our results showed that the universal chloroplast markers have low divergence (Table 2), explaining the low resolution in previous studies and highlighting the importance of developing highly divergent markers. In this study, we have identified four highly variable loci: *trnD-trnY-trnE*, *rrn16-trnI, ndhF-rpl32-trnL* and *ycf1* (Fig. 2). Of these, *rrn16-trnI* and *ycf1* have been considered divergence hotspots by Xu et al. [15], which compared six *Lagerstroemia* chloroplast genomes and identified 12 highly variable markers. Previously, *trnD-trnY-trnE* was less used in plant phylogeny. *rrn16-trnI* is located in IR regions, which are specific to the *Lagerstroemia* chloroplast genome. In general, mutation hotspots are rare in the IR region. *ndhF-rpl32-trnL* included two intergenic regions (*ndhF-rpl32* and *rpl32-trnL*), which showed the highest percentage of variable sites and the highest number of information sites (Table 2). However, there was poly A/T structure in this region, which may be regarded as low sequence quality [19, 20]. The *ycf1* locus was the most divergent marker in the *Lagerstroemia* chloroplast genome (Fig. 2) and has been broadly used for reconstructing plant phylogeny and species identification [21]. Therefore, the lineage-specific, highly variable markers developed in this study will facilitate further phylogeny reconstruction and DNA barcoding of crape myrtle species (Figure S1).

### Phylogenetics of *Lagerstroemia*

*Lagerstroemia* was a monophyletic group based on the morphology [1, 3], several chloroplast markers [22] and ITS locus [8]. De Wilde and Duyfjes [5] classified *Lagerstroemia* into four sections on the basis of the monograph by Furtado & Srisuko [1]. Several morphological features used for morphological classification of *Lagerstroemia* in previous reports, such as (1) the number of the ridges on the calyx tube, (2) the number of the ridges is the same as or twice the number of sepals, and (3) glabrous or hairy within the calyx lobes, may be observed in the same clade generated based on the molecular classification. For example, in Clade I, the 6–7 ridges on the calyx tube outside in *L. venusta* is the same as the sepal number, but each of the other two taxa (*L. speciosa* and *L. siamica*) has 12 ridges on the calyx tube outside, which is twice the number of sepals. Not ridged (*L. calyculata*), 5–6 ridges (*L. villosa*), and 12 ridges (*L. tomentosa*) are observed in Clade II. It is difficult to satisfactorily quantify the relationship between the ridge number and the sepal number when no ridge is observed. In Clade IV, *L. anhuiensis* has hairs within calyx lobes, but it is glabrous within calyx lobes in *L. guilingensis*, *L. caudata*, *L. glabra* and *L. indica*.

Molecular markers, such as AFLP, SSRs [23], were used to distinguish the cultivars of *Lagerstroemia* species, such as *L. indica*, *L. subcostata*, *L. limii* and *L. fauriei*. However, the genetic background of the cultivars was unclear, and these markers were not informative to infer the relationship of those species. The chloroplast genome has become an efficient option for increasing plant phylogenomics at multiple taxonomic levels during the past years [24–29]. We had used the chloroplast genome data to infer phylogenetic relationships of six *Lagerstroemia* species, and discovered that the chloroplast genome sequences had effective information to infer the phylogeny of this genus [15].

**Fig. 3:**
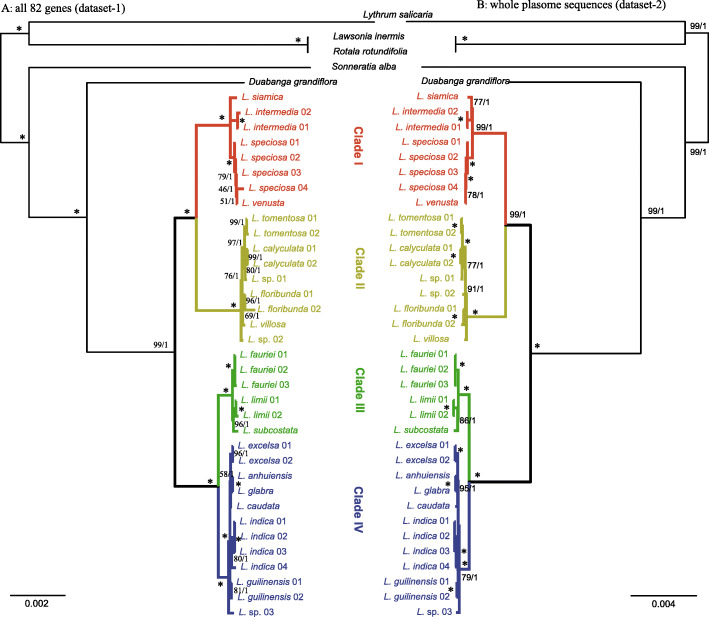
Molecular phylogeny of *Lagerstroemia* from ML (maximum likelihood) and BI (Bayesian inference) analyses using different data sets. **A**. Eighty-three coding genes (dataset-1); **B**. the chloroplast genome sequences (dataset-2). Maximum likelihood bootstrap values (BS) and posterior probabilities (PP) are shown at nodes. Branches with * indicate 100% BS and a PP of 1.0

**Fig. 4:**
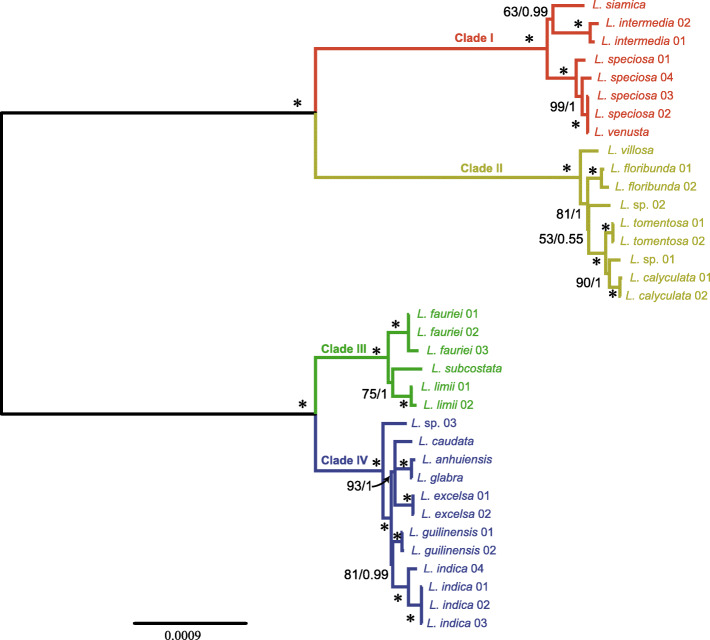
Molecular phylogeny of *Lagerstroemia *resulting from ML and BI analyses using whole chloroplast genome sequences (dataset-3). Maximum likelihood bootstrap values (BS) and posterior probabilities (PP) are shown at nodes. Branches with * indicate 100% BS and a PP of 1.0

In this study, we recovered a well-supported and species-level relationship of *Lagerstroemia* using six different chloroplast genome datasets. It provided strong support for the monophyly of *Lagerstroemia*, sister to *Duabanga*, and recovered four major clades (Figs. 3 and 4). However, the four clade classifications were different from the morphological classification of the genus [1]. For example, *L. speciosa, L. limii*, and *L. glabra* were in the section *Adambea*, the molecular results showed *L. speciosa* was in the clade 1, *L. limii* in the clade 3, and *L. glabra* in the clade 4, respectively.

In clade I, *L. venusta* was a hexaploid species [11] and fell within the *L. speciosa* phylogenetically (Figs. 3 and 4). We inferred that *L. venusta* might be an allohexaploid species and its female parent was *L. speciosa*. The branch length was short in most terminal nodes, which showed *Lagerstroemia* may be undergone a rapid radiation [30, 31]. The phylogenomics of Myrtales based on 66 protein-coding genes showed the 14 *Lagerstroemia* species formed four clades [17]. However, the relationship of *Lagerstroemia* was inconsistent with this study. The difference might be caused by the longer branch length of *L. intermedia* [17] which affected the topology of the phylogenetic tree. We used the same dataset to infer a similar tree as this study. Further investigations, including extended sampling, more morphological analysis and additional nuclear markers, are needed to insight the evolution of *Lagerstroemia.*

### Divergence time of *Lagerstroemia*

**Fig. 5:**
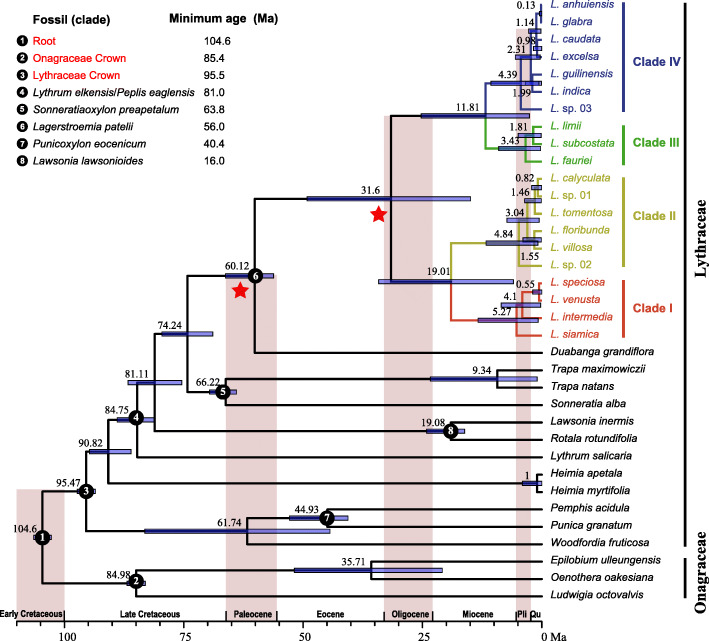
Maximum clade credibility (MCC) tree of Lythraceae obtained from BEAST analysis. Mean divergence time estimates are shown with 95% highest posterior density (HPD; blue bars). Black circles indicate the eight calibration points

The fossil record of the *Lagerstroemia* consists of leaf impressions, wood, and pollen [18]. According to the fossil record, the oldest confirmed evidence of the *Lagerstroemia* is a leaf impression of *L. patelii* from India, which was dated as early Eocene or late Paleocene/Thanetian in age (~ 56 Ma) [32, 33]. The oldest occurrence of accepted *Lagerstroemia* pollen is from the middle Eocene of Central Java [34]. Those records indicated the origin time of *Lagerstroemia* was earlier than 56 Ma. Our data also support a late Paleocene origin (~ 60 Ma, Fig. 5; Table 2).

There were a number of putative fossil *Lagerstroemia* leaves and wood in the middle Miocene [18]. For example, the leaf species of *L. mioparviflora*, *L. eomicrocarpa* and *L. siwalica* were described from Nepal [35, 36], and *L. jamraniensis* was from the Kathgodam area [37]. The wood fossil record of *Lagerstroemia* is used as the form genus *Lagerstroemioxylon* Mädler. The wood is recorded from Sumatra (*Lagerstroemioxylon eoflosreginum*)[38] and Myanmar (*Lagerstroemioxylon irrawaddiensis*) [39] and is widely encountered in India at several localities (*Lagerstroemioxylon arcotense*, *Lagerstroemioxylon deomaliensis*, *Lagerstroemioxylon eoflosreginum*) [18, 40]. Those fossil records suggest that *Lagerstroemia* was common and somewhat diverse in the wet subtropical forests of the Indian subcontinent in the middle Miocene. The phylogeny and dating analyses demonstrate a similar pattern of this genus divergence into four clades during the Miocene ~ 20 Ma. Diversification with *Lagerstroemia* occurred in the Pleistocene ~ 5.3 Ma, and at this time, this genus is present and persists in Japan [18, 41].

## Conclusions

In this study, we report 20 newly sequenced chloroplast genomes of the genus *Lagerstroemia*. The overall genomic structure, including gene number and gene order, was well-conserved. The relationship and divergence times of *Lagerstroemia* were revealed using complete chloroplast genome sequence data. Four clades were found in this genus. Greater taxon sampling is necessary to determine the number of species, morphological characteristics, evolution and biogeography. Our study showed that the chloroplast genome data will provide adequate information for resolving the phylogenetic relationships in this difficult-to-characterize genus.

## Methods

### Plant materials, genomic DNA extraction and sequencing

According to the morphological classification, the *Lagerstroemia* was classified into four sections and eight subsections [1]. In order to infer the framework of the phylogenetic relationship, we sampled 20 individuals of 17 described species, which represented all the four sections and six of eight subsections. The materials were obtained from the field, botanical gardens and the herbarium of the Institute of Botany, Chinese Academy of Sciences (PE, Table S1). Three crape myrtle samples could not be accurately identified morphologically because of the lack of morphological characters. In addition to the newly collected material for DNA sequencing, publicly available complete chloroplast genome sequences (15 accessions, Table S1) of *Lagerstroemia* were also included in this analysis.

Total genomic DNA was extracted from silica-dried leave tissues of living plants and herbarium specimens of this genus following the modified CTAB DNA extraction protocol [42]. The DNA from silica-dried tissue was fragmented to construct 350-bp insert libraries, and the DNA from the herbarium material was constructed using 150-bp insert libraries according to the manufacturer’s manual (Illumina Inc., San Diego, CA, USA) and was then used for sequencing. Paired-end sequencing was performed on an Illumina HiSeq X-ten at Novogene in Tianjin, China, yielding approximately 4 Gb of high-quality 150-bp paired-end reads per sample.

### Chloroplast genome assembly, annotation, and comparative analyses

A four-step approach was employed to assemble the chloroplast genome. First, adaptors were removed, and low-quality sequences were trimmed using Trimmomatic 0.39 [43] with the following parameters: LEADING = 20, TRAILING = 20, SLIDINGWINDOW = 4:15, MINLEN = 36 and AVGQUAL = 20. Second, remaining high-quality reads were assembled de novo into contigs using SPAdes 3.6.1 [44]. Third, chloroplast genome sequence contigs were selected from the initial assembly by performing a BLAST search using the *L. subcostata* chloroplast genome sequence as a reference (GenBank accession number: KF572029). The selected contigs from chloroplast genomes were further assembled using Sequencher 5.4.5 (http://www.genecodes.com). Fourth, Geneious 11.1.2 was used to map all reads to the assembled chloroplast genome sequence to check the four junctions between the inverted repeats (IRs) and the small single-copy (SSC)/large single-copy (LSC) regions.

Chloroplast genome sequences were annotated using Plann [45] and, missing or incorrect genes were checked in Sequin. Physical maps of the circular chloroplast genomes were visualized with OGDRAW [46]. To assess sequence divergence and to explore highly variable chloroplast markers, nucleotide diversity (π) was calculated by sliding window analysis using DnaSP v6 [47], and nucleotide substitutions and p-distance were calculated using MEGA 7.0 [48].

### Alignment and data matrix construction

The sequence alignments were constructed with MAFFT v7 [49]. All alignments were visually inspected with MEGA 7.0 [48] and manually adjusted where needed. To access the phylogenetic effects of the different regions in the chloroplast genome, we created six datasets based on different chloroplast genome regions or using different outgroups. All 78 protein-coding genes and four rRNA genes were extracted from the GenBank-formatted files containing all chloroplast genomes using Python scripts. Those 82 genes were combined into a concatenated dataset as dataset-1. Dataset-2 included 35 whole chloroplast genome sequences of *Lagerstroemia* and five other species of Lythraceae as outgroups (*Lythrum salicaria*, *Lawsonia inermis*, *Rotala rotundifolia*, *Sonneratia alba*, and *Duabanga grandiflora*). Ambiguous alignment regions were trimmed using Gblocks 0.91b [50] implemented in Phylosuite v1.1 [51]. In addition, the third to sixth datasets only included 35 samples of *Lagerstroemia*, which were from the complete chloroplast genomes, LSC region, IR region, and SSC region, respectively.

### Phylogenetic analyses

We used maximum likelihood (ML) and Bayesian inference (BI) methods for phylogenetic analyses. The datasets were unpartitioned, and the best-fit model was determined by ModelFinder [52]. Maximum likelihood analyses were run with RAxML v.8.1.24 [53]. RAxML searches were made with 500 randomized maximum parsimony starting trees, and RAxML was run again under the same conditions executing 1,000 nonparametric bootstrap replicates to assess the branch support.

BI was run with Mrbayes v3.2 [54]. Two independent Markov Chain Monte Carlo (MCMC) analyses were performed, each with four chains (three heated and one cold) for 20 million generations with sampling of every 100th tree. Each chain started with a random tree, and the first 25 % sampled generations were discarded as burn-in to construct a majority-rule consensus tree and to estimate posterior probabilities (PP). Stationarity was considered to be reached when the average standard deviation of split frequencies was < 0.01.

### Fossil priors and BEAST analyses

We used BEAST v2.5.1 [55] to estimate the divergence times using dataset-1 and added seven Lythraceae species and three Onagraceae species to accommodate all available fossil calibrations. This dataset was calibrated using five reliably dated fossils. The pollen of *Lythrum elkensis* Grimsson et al./*Peplis eaglensis* Grimsson et al. was recently described from the Late Cretaceous early Campanian (82 − 81 Ma) Eagle Formation at Elk Basin, Wyoming, USA [18]. This fossilized pollen was used to offset for the crown of the two lineages. *Sonneratiaoxylon preapetalum* Awasthi was fossil wood of *Sonneratia* [56] from the early Paleocene of India (Danian, 67.3 − 63.8 Ma) and was used to calibrate the most recent common ancestor (TMRCA) of *Sonneratia* and *Trapa* to > 63.8 Ma. We also used the oldest fossil accepted as *Punica*, which was wood of *Punicoxylon eocenicum* Privé-Gill from the middle Eocene (48.6 − 40.4 Ma) of Paris [18], and the seed of *Lawsonia lawsonioides* (Menzel) Mai. [57] from the middle Miocene (16 Ma ago) as conservative offsets on the stem nodes of *Punica* and *Lawsonia*, respectively. The oldest confirmed fossil of *Lagerstroemia patelii* Lakhanpal & Guleria, from the late Paleocene/Eocene (ca. 56 Ma) was used to calibrate the stem age of this genus to > 56 Ma [18, 58]. Each of the five fossil priors (*Lythrum elkensis*/*Peplis eaglensis*, *Sonneratiaoxylon preapetalum*, *Punicoxylon eocenicum*, *Lawsonia lawsonioides*, and *Lagerstroemia patelii*) was given a lognormal distribution with offset values as specified (i.e., 81.0, 63.8, 40.4, 16.0, and 56.0 Ma, respectively), and with a mean of 1.5 and a standard deviation of 1, allowing for the possibility that these nodes are considerably older than the fossils themselves. In addition to these fossil priors, we also used three secondary priors. Based on the average value obtained by Berger et al. [59] in a calibrated analysis, three priors were used: (1) the average age of TMRCA of Lythraceae and Onagraceae (the root of the tree) was 104.6 Ma; (2) the crown age of Onagraceae was 85.4 Ma; and (3) the crown age of Lythraceae was 95.5 Ma. Each secondary prior was placed under normal distribution with a standard deviation of 1.

To assess possible calibration incongruence, we ran twelve analyses with calibration combinations (Table 2). The twelve analyses were run with uncorrelated lognormal distribution (UCLD) relaxed molecular clock models to account for rate variability among lineages, the Yule speciation model and 100,000,000 generations with the MCMC method, sampling trees every 10,000 generations. The stationary phase was examined through Tracer 1.6 [60] to evaluate convergence and to ensure sufficient and effective sample size (ESS) for all parameters surpassing 200. A burn-in of 10 % generations was discarded, and TreeAnnotator v2.4.7 was used to produce a Maximum Clade Credibility tree.

## Supplementary Information

**Additional file 1: Table S1. T**axa included in the present study. Collection locality and voucher information are provided for newly sequenced samples.

**Additional file 2: Figure S1. **ML tree for *Lagerstroemia* using combined three universal plant DNA barcodes and four highly variable regions.

**Additional file 3: Figure S2. **Molecular phylogeny of *Lagerstroemia* resulting from ML (maximum likelihood) and BI (Bayesian inference) analyses using LSC regions (dataset-4). Maximum likelihood bootstrap values (BS) and posterior probabilities (PP) are shown at nodes. Branches with * indicate 100 % BS and a PP of 1.0.

**Additional file 4: Figure S3. **Molecular phylogeny of *Lagerstroemia* resulting from ML (maximum likelihood) and BI (Bayesian inference) analyses using IR regions (dataset-5). Maximum likelihood bootstrap values (BS) and posterior probabilities (PP) are shown at nodes. Branches with * indicate 100 % BS and a PP of 1.0.

**Additional file 5: Figure S4. **Molecular phylogeny of *Lagerstroemia* resulting from ML (maximum likelihood) and BI (Bayesian inference) analyses using SSC regions (dataset-6). Maximum likelihood bootstrap values (BS) and posterior probabilities (PP) are shown at nodes. Branches with * indicate 100 % BS and a PP of 1.0.

## Data Availability

The chloroplast genome of *Lagerstroemia* under study is deposited in the GenBank database under the following accession numbers: MT019844 - MT019863. The other sequences used in this study were downloaded from the NCBI.

## Abbreviations

BI: Bayesian Inference
bp: Base pairs
Gb: Gigabases
LSC: Long single copy
Ma: Million years ago
MCMC: Markov chain Monte Carlo
ML: Maximum likelihood
NCBI: National Center for Biotechnology Information
NGS: Next generation sequencing
π: Nucleotide diversity
rRNA: Ribosomal RNA
SSC: Short single copy
SSR: Simple sequence repeat
tRNA: Transfer RNA
