# Supplementary figures and images for: Chloroplast phylogenomics and divergence times of Lagerstroemia (Lythraceae)
Source: BMC Genomics. 2021 Jun 9;22:434. doi: 10.1186/s12864-021-07769-x (PMC8191006; doi:10.1186/s12864-021-07769-x)

a: Three universal DNA barcodes

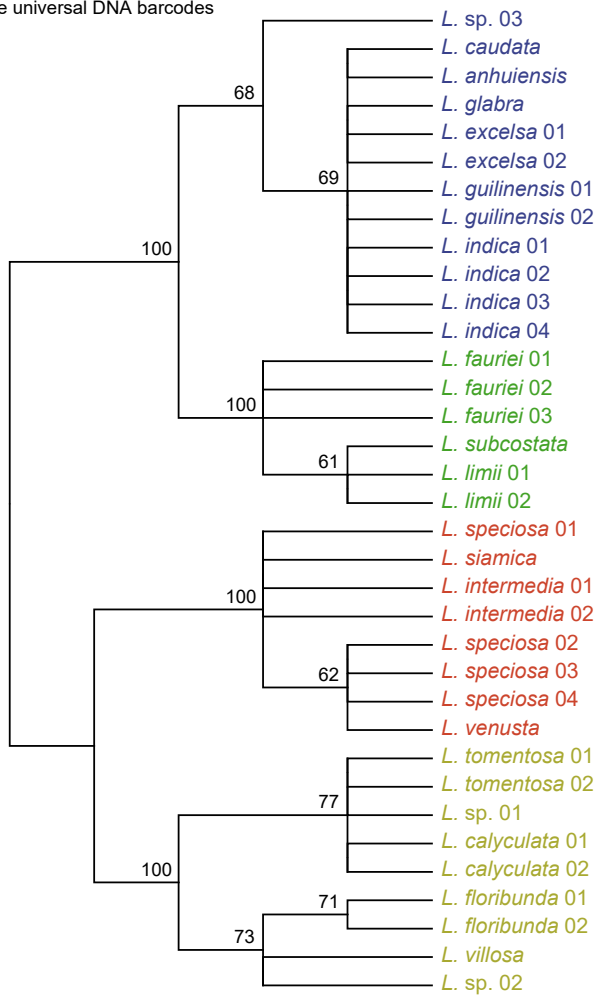

b: Four highly variable markers

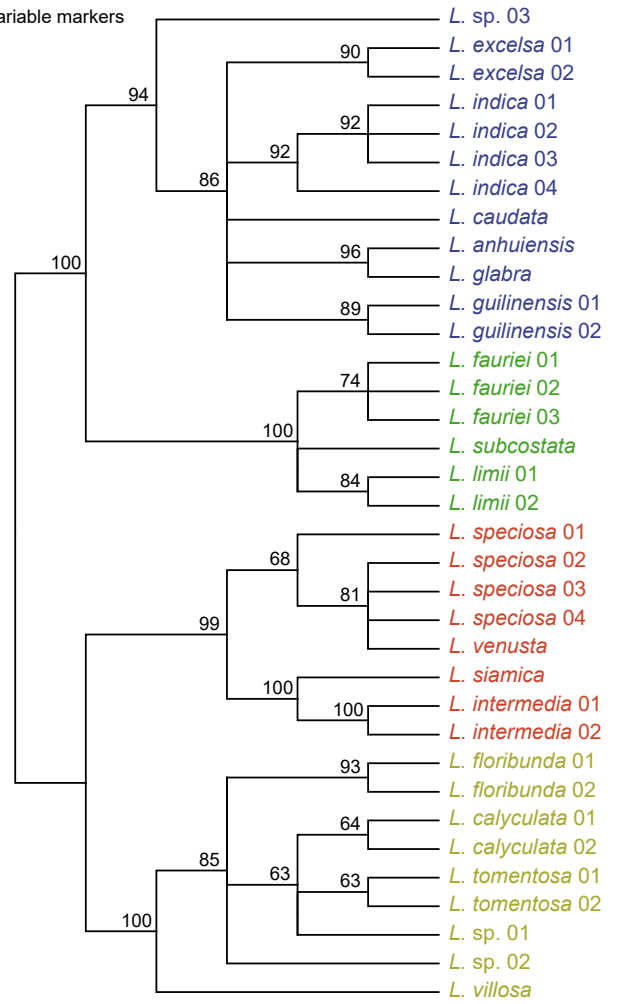

Supplement: Supplementary file 2 — Additional file 2: Figure S1. ML tree for Lagerstroemia using combined three universal plant DNA barcodes and four highly variable regions. [file 12864_2021_7769_MOESM2_ESM.pdf]

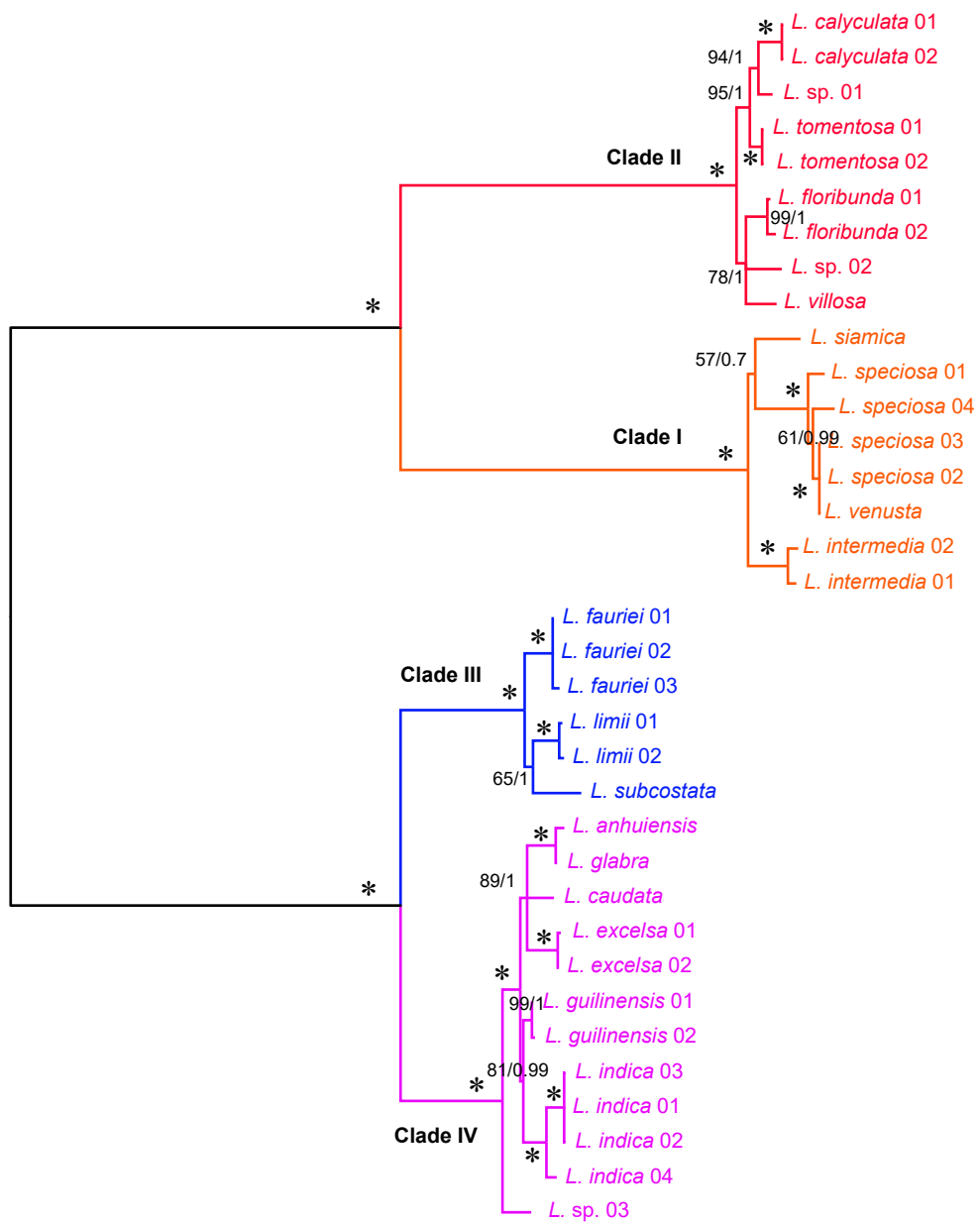

9.0E-4

Supplement: Supplementary file 3 — Additional file 3: Figure S2. Molecular phylogeny of Lagerstroemia resulting from ML (maximum likelihood) and BI (Bayesian inference) analyses using LSC regions (dataset-4). Maximum likelihood bootstrap values (BS) and posterior probabilities (PP) are shown at nodes. Branches with * indicate 100 % BS and a PP of 1.0. [file 12864_2021_7769_MOESM3_ESM.pdf]

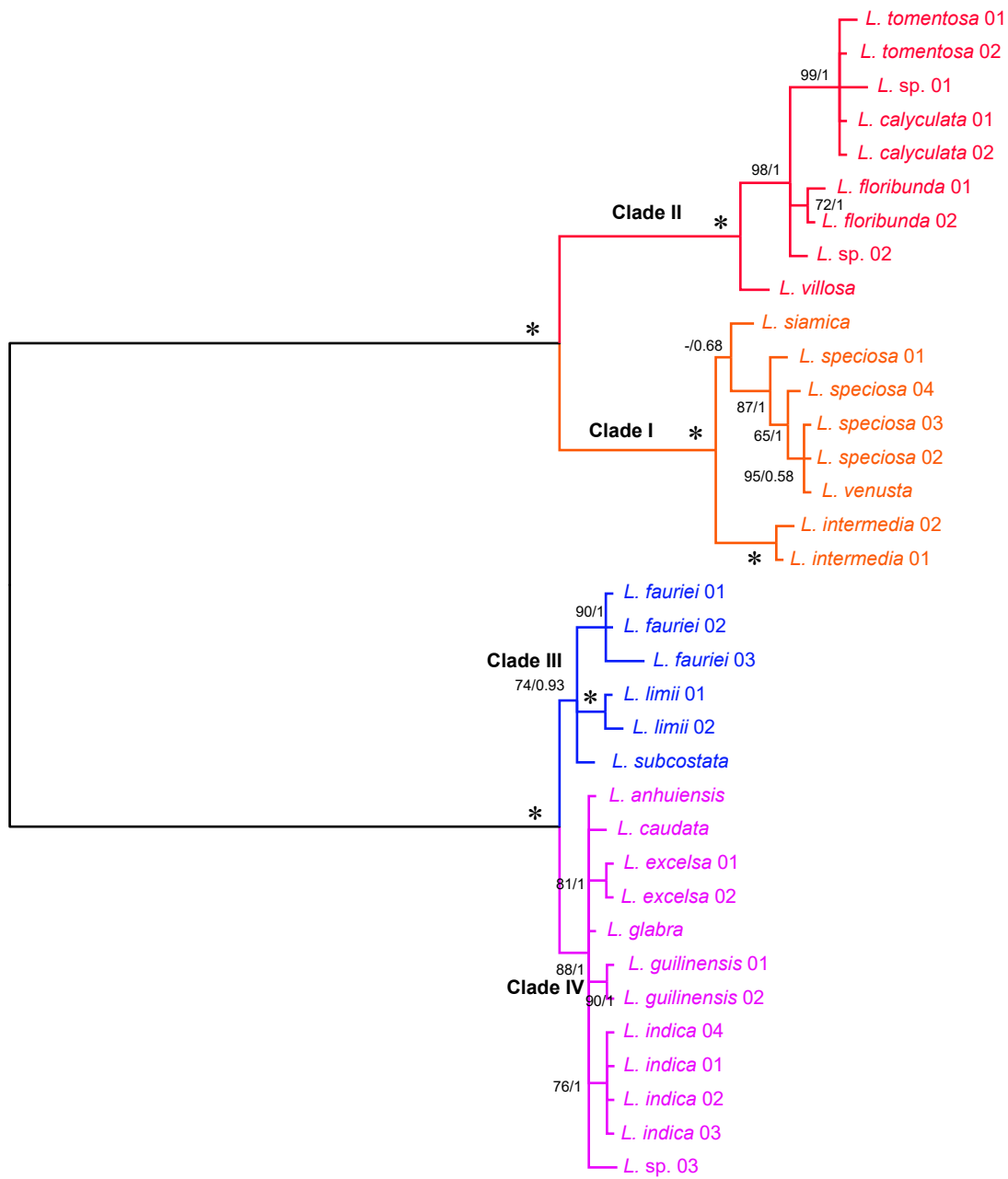

Supplement: Supplementary file 4 — Additional file 4: Figure S3. Molecular phylogeny of Lagerstroemia resulting from ML (maximum likelihood) and BI (Bayesian inference) analyses using IR regions (dataset-5). Maximum likelihood bootstrap values (BS) and posterior probabilities (PP) are shown at nodes. Branches with * indicate 100 % BS and a PP of 1.0. [file 12864_2021_7769_MOESM4_ESM.pdf]

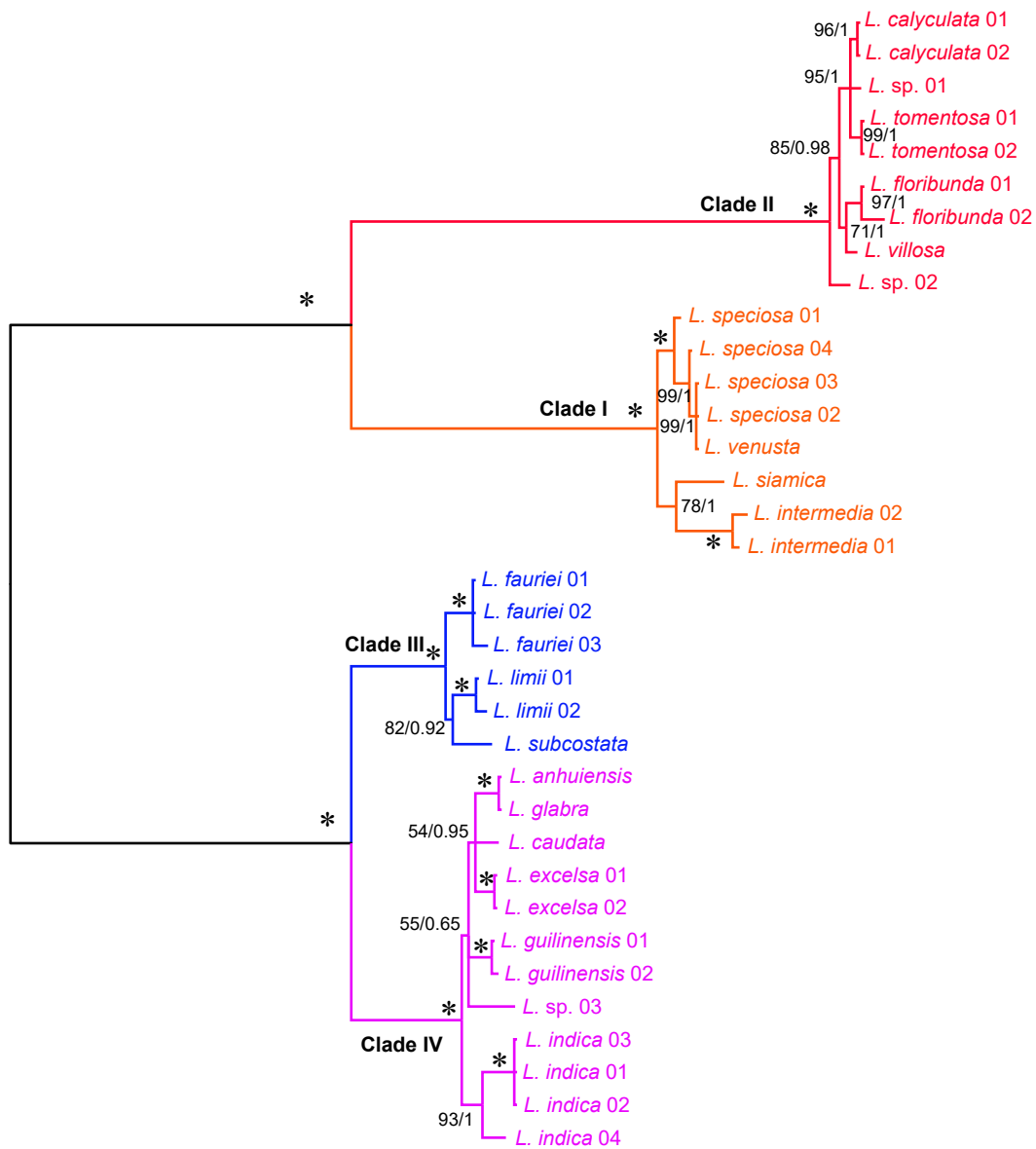

0.002

Supplement: Supplementary file 5 — Additional file 5: Figure S4. Molecular phylogeny of Lagerstroemia resulting from ML (maximum likelihood) and BI (Bayesian inference) analyses using SSC regions (dataset-6). Maximum likelihood bootstrap values (BS) and posterior probabilities (PP) are shown at nodes. Branches with * indicate 100 % BS and a PP of 1.0. [file 12864_2021_7769_MOESM5_ESM.pdf]
